# Three-dimensional finite element analysis of the effects of different distal-end aligner designs on maxillary premolar distalization

**DOI:** 10.3389/fbioe.2026.1775597

**Published:** 2026-02-26

**Authors:** Zhijie Yang, Xiangyu Ma, Yang Liu, Tao Xu, Tianwei Shang, Yue Liu, Yifan Zhang, Cunhui Fan

**Affiliations:** 1 Department of Orthodontics, The Affiliated Hospital of Qingdao University, Qingdao, Shandong, China; 2 School of Stomatology, Qingdao University, Qingdao, Shandong, China; 3 Department of Stomatology, Qingdao Huangdao District Hospital of Traditional Chinese Medicine, Qingdao, Shandong, China

**Keywords:** clear aligner appliance, finite element analysis, molar distalization, orthodontics, tooth movement

## Abstract

**Background:**

Sequential distalization of the maxillary dentition is widely used to gain space for crowding relief, molar relationship correction, and facial profile improvement. Using three-dimensional finite element analysis, we evaluated the biomechanical impact of different distal-end clear aligner designs during premolar distalization in maxillary sequential distalization, with the aim of reducing mesial relapse of teeth that had already reached their planned positions and improving overall distalization efficiency.

**Material and methods:**

Two initial models were constructed: In Model A, both maxillary molars were distalized by 2 mm to reach the planned positions. In Model B, both maxillary molars and the maxillary second premolar were distalized by 2 mm to reach the planned positions. Four aligner configurations were defined in Model A: A0, control (conventional design); A1, distal coverage removed at U7; A2, distal coverage removed at U6; and A3, distal coverage removed at U7 and U6. Five aligner configurations were defined in Model B: B0, control (conventional design); B1, distal coverage removed at U7; B2, distal coverage removed at U5 and U6; B3, distal coverage removed only at U5; and B4, distal coverage removed at U7, U6, and U5. We quantified tooth displacement, space-closure component ratios, and periodontal ligament (PDL) equivalent stress under each condition.

**Results:**

For Model A, the contribution of U5 distal displacement to U5–U6 space closure was 68.08%, 66.48%, 70.44%, and 92.01% in A0–A3, respectively, and was highest in A3. In Model B, the contribution of U4 distal displacement to U4–U5 space closure was 69.22%, 68.15%, 70.55%, 70.47%, and 81.19% in B0–B4, respectively, and was highest in B4.

**Conclusion:**

During second premolar distalization in maxillary sequential distalization, removing distal-portion coverage at U7 and U6 effectively reduced mesial relapse of already distalized molars, thereby protecting posterior anchorage. Similarly, during first premolar distalization, to reduce relapse of distalized teeth, distal-portion coverage should be removed at U7, U6, and U5.

## 1 Introduction

Clear aligners have become increasingly popular in orthodontic treatment as an alternative to fixed appliances because of their esthetics, comfort, and reduced chairside time (Gu et al., 2017). For Angle Class II patients, especially adults, clinicians often rely on dentoalveolar tooth movement alone to improve the molar relationship and reduce maxillary protrusion (Raghis et al., 2022). Maxillary sequential distalization refers to sequential distal movement of the molars and premolars to gain space and is commonly used to relieve crowding, correct molar relationships, and improve the facial profile (Bolla et al., 2002). With conventional fixed appliances, sequential distalization is often accompanied by distal tipping of the maxillary molars and labial proclination of the maxillary anterior teeth (Al-Thomali et al., 2017). Distal tipping of the maxillary molars may induce clockwise rotation of the occlusal plane and increase lower facial height, potentially worsening the facial profile in Class II patients (Fortini et al., 2004). In contrast, because clear aligners cover the entire crown surface, they may distribute forces more uniformly than conventional distalizing appliances and may theoretically facilitate more bodily tooth movement (Karras et al., 2021). Caruso et al. retrospectively assessed molar distalization with clear aligners using lateral cephalograms and suggested that aligners can provide better control of vertical tooth movement, which may benefit open-bite or hyperdivergent patients (Caruso et al., 2019). Simon et al. reported that maxillary molar distalization was one of the most effective movements, with an effectiveness of up to 87% (Simon et al., 2014). Rever et al. reported that when the maxillary dentition was distalized by 2–3 mm, following a 50% sequential distalization protocol combined with Class II elastics resulted in limited mesiodistal tipping and more bodily movement (Ravera et al., 2016). Using palatal rugae superimposition immediately after distalization, Badr Sultan Saif et al. reported a mean maxillary molar distalization efficiency of 73.8% (Saif et al., 2022). Therefore, sequential distalization has been regarded as a strength of clear aligners and one of the most predictable tooth movements achievable with aligners (Rossini et al., 2015).

However, clinical experience suggests that the achieved maxillary molar distalization with clear aligners is often less than predicted and may be accompanied by side effects such as incisor proclination and increased upper lip prominence (Mariani et al., 2014). More recent evidence suggests that the final achievement of molar distalization is far lower than earlier reports. Liu et al. performed voxel-based superimposition of pre- and post-treatment CBCT scans in 30 patients and found that distalization efficiency of the maxillary posterior teeth with clear aligners was only 36.2%–43.9% (Liu et al., 2024). Li et al. investigated full stage maxillary distalization and found that the final achievement rates were only 36.48% for the first molar and 41.94% for the second molar (Li et al., 2023). Qiang et al., using CBCT superimposition before and after treatment, reported final crown distalization efficiencies of 5.58%, 10.13%, 19.21%, and 31.06% for the first premolar, second premolar, first molar, and second molar, respectively, and final root distalization efficiencies of 19.30%, 34.22%, 31.33%, and 37.89%, concluding that sequential distalization with clear aligners is feasible but far less achievable than expected (Qiang et al., 2025). Collectively, these findings suggest that the predictability of sequential distalization may be lower than previously assumed.

Two factors may contribute to the reduced overall efficiency of full-arch sequential distalization: loss of anterior anchorage due to incisor proclination and loss of posterior anchorage due to mesial relapse of already distalized molars.

Clinically, the efficiency of molar distalization is best interpreted in terms of its contribution to patient-relevant outcomes, particularly the improvement of anterior protrusion and resolution of crowding. Therefore, the clinically meaningful outcome is preservation of the space created by posterior distalization so that it can be effectively utilized for anterior tooth movement. In other words, preserving the space created by molar and premolar distalization is more important and clinically meaningful. In aligner treatment, forces are generated by the mismatch between aligner and current tooth position. During distalization of the first molar, the mesial aligner segment effectively elongates while the distal segment shortens. The second molar is subjected to a mesial force from this shortening, leading to mesial movement that encroaches on the space between the first and second molars, which causes mesial relapse of the second molar (Ma et al., 2025). A similar relapse may also occur during premolar distalization stage. Mesial relapse of already distalized teeth can substantially reduce effective utilization of the space gained.

In our prior study, Ma introduced a distal non-coverage design for U7 (maxillary second molar) and reported, based on 3D finite element simulations, that it reduced mesial relapse of U7 during U6 (maxillary first molar) distalization, supporting improved sequential molar distalization efficiency (Ma et al., 2025). Using 3D finite element analysis, Zhang (Zhang et al., 2025) found that Class II elastics had limited ability to protect second molar anchorage during sequential molar distalization. Distal non-coverage design for U7 was reported to reduce second molar anchorage loss. Class II elastics may still be required to protect anterior anchorage. Therefore, appropriate strategies to prevent mesial relapse of already distalized teeth and to maintain the gained space are essential. However, limited evidence specifically addresses how to reduce mesial relapse of already distalized molars.

Three-dimensional finite element analysis is a computational approach that, among methods for investigating clear aligner biomechanics, can evaluate stress distribution within periodontal tissues with good reproducibility and comparability (Cattaneo and Cornelis, 2021). Because orthodontic treatment is time-consuming, clinically meaningful biomechanical effects are difficult to obtain in the short term. Accordingly, 3D finite element methods have been increasingly applied in orthodontics to study the biomechanics of clear aligners (Elshazly et al., 2023).

Accordingly, this study used three-dimensional finite element analysis to evaluate different distal-end aligner designs aimed at reducing relapse of already distalized teeth during maxillary sequential distalization. We hypothesized that distal non-coverage would improve posterior anchorage preservation by reducing mesial relapse without compromising the efficiency of ongoing staged distalization.

## 2 Materials and methods

### 2.1 Subjects and data collection

Cone-beam computed tomography (CBCT) data from an adult female volunteer treated at the Department of Orthodontics of Qingdao University Affiliated Hospital were used as the source data. The volunteer presented with a complete permanent dentition and normal tooth morphology; no systemic medical history; no history of craniofacial trauma, periodontal disease, temporomandibular joint disorders, or previous orthodontic treatment; skeletal Class II and Angle Class II malocclusion; and Grade I increased overjet and Grade I deep overbite. We have obtained informed consent from the volunteer, all methods were performed in accordance with the Declaration of Helsinki. The study was approved by the Ethics Committee of the Affiliated Hospital of Qingdao University (approval No. QYFY-WZLL-30878).

### 2.2 Model construction

The DICOM-format CBCT data were imported into Mimics Medical 21.0 (Materialise, Belgium), where masks were created using threshold segmentation, and the maxilla and teeth were segmented using a region-growing algorithm to generate separate 3D models, which were exported as STL files.

**FIGURE 1:**
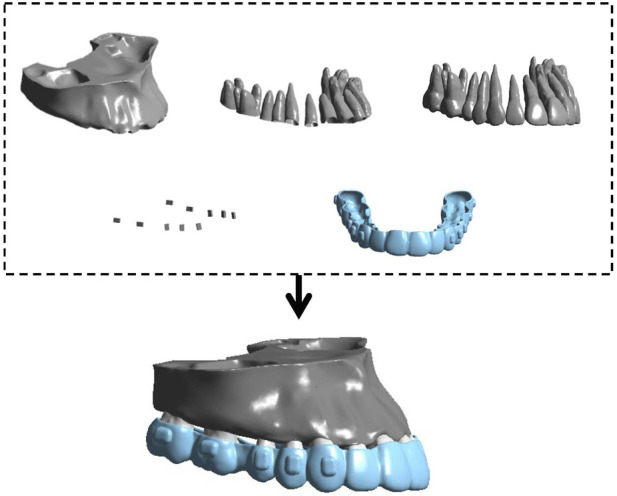
Finite element models, consisting of maxillary bone, PDLs, maxillary dentitions, clear attachments and aligners.

The STL files were then imported into Geomagic Wrap 17.0 (3D Systems, United States of America) for surface smoothing and surface fitting, and a 3D periodontal ligament (PDL) model was generated by offsetting the root surface of each maxillary tooth by 0.25 mm (Mao et al., 2023). In SolidWorks (Dassault Systems, France), horizontal rectangular attachments were created on the buccal surfaces from the second molar to the first molar, and vertical rectangular attachments were created on the buccal surfaces from the second premolar to the canine using the boss-extrude feature (Figure 1). Conventional vertical and horizontal rectangular attachments were designed according to the manufacturer’s suggstions (Mao et al., 2023). The attachment configuration was standardized across all design groups to isolate the biomechanical effect of different distal-end aligner designs. In this study, a V-pattern sequential distalization protocol was simulated. Distalization began with the maxillary second molars; once the second molars had achieved approximately one-half of the planned distalization, the maxillary first molars were initiated to move distally, followed sequentially by the premolars, and finally en-masse retraction of the four incisors to complete the treatment plan (Mao et al., 2023). The maxilla, teeth, PDL, and attachments were assembled. Two modles were established: Model A: the second and first molars were distalized by 2 mm, representing the stage of the second premolar distalization. Model B: the second molar, first molar, and second premolar were distalized by 2 mm, representing the stage of the first premolar distalization. Because the mesial force on teeth that have already been distalized mainly originates from aligner coverage on their distal surfaces, the distal end of the clear aligner was modified and grouped as follows. Distal non-coverage of the aligner was defined as removal of all aligner coverage from the distal surface, including the gingival extension to the gingival margin, bounded by the disto-occlusal, disto-buccal, and disto-lingual line angles.

**FIGURE 2:**
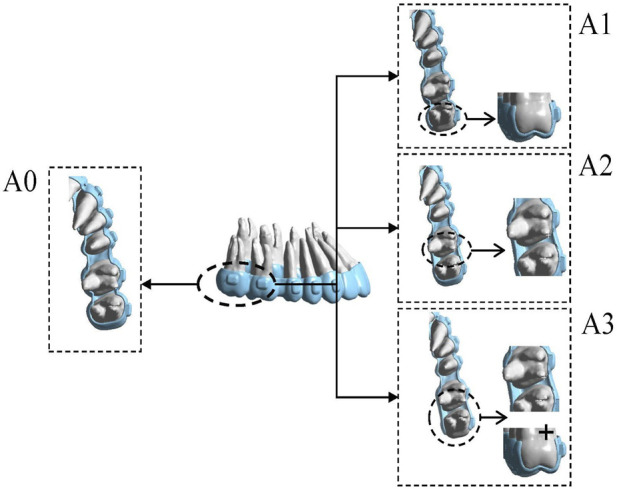
The sets of groups were designed on the basis of different distal end of the clear aligner. In Modle A, four configurations were established: A0, control (conventional aligner design); A1, distal coverage removed at U7; A2, distal coverage removed at U6; and A3, distal coverage removed at both U7 and U6. Distal non-coverage of the aligner was defined as removal of all aligner coverage from the distal surface, including the gingival extension to the gingival margin, bounded by the disto-occlusal, disto-buccal, and disto-lingual line angles.

**FIGURE 3:**
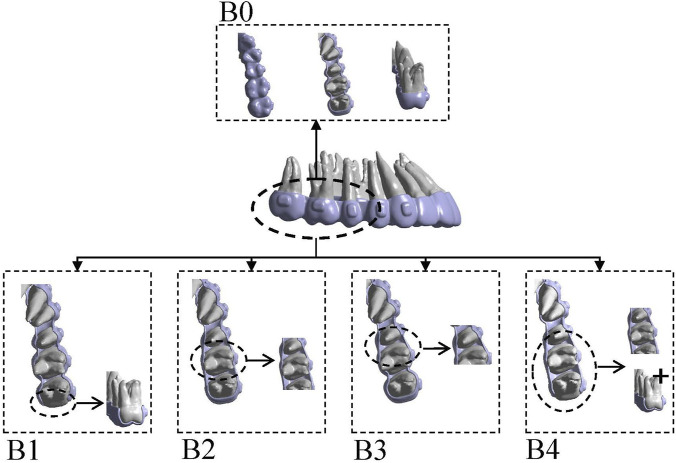
The sets of groups were designed on the basis of different distal end of the clear aligner. In Model B, five configurations were established: B0, control (conventional aligner design); B1, distal coverage removed at U7; B2, distal coverage removed at U5 and U6; B3, distal coverage removed only at U5; and B4, distal coverage removed at U7, U6, and U5. Distal non-coverage of the aligner was defined as removal of all aligner coverage from the distal surface, including the gingival extension to the gingival margin, bounded by the disto-occlusal, disto-buccal, and disto-lingual line angles.

In Model A (second premolar distalization after two molars had reached the planned position), four groups were established: A0, control (conventional aligner design); A1, distal coverage removed at U7; A2, distal coverage removed at U6; and A3, distal coverage removed at both U7 and U6 (Figure 2). In Model B (first premolar distalization after the molars and second premolar had reached the planned positions), five groups were established: B0, control (conventional aligner design); B1, distal coverage removed at U7; B2, distal coverage removed at U5 and U6; B3, distal coverage removed only at U5; and B4, distal coverage removed at U7, U6, and U5 (Figure 3). Staging was set at 0.25 mm per aligner, consistent with mainstream clear aligner (CA) design systems, such as ClinCheck for Invisalign (Align Technology, Santa Clara, CA, United States of America) and iOrtho for Angelalign (Angelalign Technology, Shanghai, China) (Ravera et al., 2016; Gao et al., 2024). In Model A, the second premolar was moved distally by 0.25 mm to generate aligners A0–A3. In Model B, the first premolar was moved distally by 0.25 mm to generate aligners B0–B4.

### 2.3 Material properties

Based on previous studies, all structures were simplified as homogeneous, isotropic, linear elastic materials, and the material properties (Poisson’s ratio and Young’s modulus) were assigned according to the literature (Gomez et al., 2015; Cortona et al., 2020; Cheng et al., 2022) (Table 1). Notably, the aligner was modeled as a homogeneous, isotropic, linear elastic material to represent the initial force system immediately after aligner insertion.

**TABLE 1:** Material properties.

Material	Young’s modulus (MPa)	Poisson’s ratio
Tooth	19,600	0.3
PDL	0.67	0.45
Alveolar bone	13,700	0.3
Clear aligner	528	0.36
Attachments	12,500	0.36

### 2.4 Meshing

Each aligner was assembled with the initial dentition, and the assembled models were imported into ANSYS 2021R2 (ANSYS, United States of America) for meshing; the mesh size was set to 1.0 mm for the maxilla and 0.3 mm for the teeth, periodontal ligament, attachments, and aligner. To balance computational efficiency and accuracy, second-order tetrahedral elements were used. The numbers of elements and nodes for each group are shown in Table 2.

**TABLE 2:** Nodes and elements.

Item	A0	A1	A2	A3	B0	B1	B2	B3	B4
Nodes	1,347,388	1,338,127	1,364,022	1,332,011	1,652,251	1,651,321	1,625,270	1,634,578	2,317,192
Elements	844,213	837,027	852,464	834,771	1,048,789	1,046,754	1,031,821	1,037,102	1,533,650

### 2.5 Boundary conditions and loading

The superior surface of the maxilla was fixed. Surface-to-surface bonded contact was defined between the root–PDL–maxilla and between the crown and attachments; friction contact was defined between tooth contact surfaces; and frictional contact was defined between the crowns and the aligner and between the attachments and the aligner, with a friction coefficient of 0.2 (Liu et al., 2023). Loads were applied through the mismatch between the aligner and the initial dentition.

### 2.6 Data extraction

**FIGURE 4:**
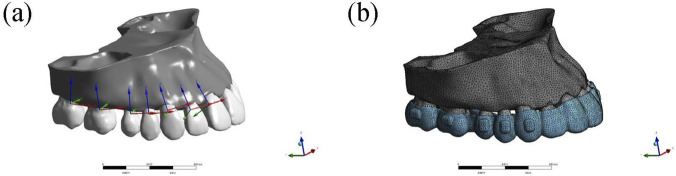
**(a)** The global and local coordinate systems. **(b)** The mesh figure.

The outcomes were tooth displacement and equivalent stress in the periodontal ligament after loading each aligner condition; because the dental arch is bilaterally symmetric, only the right maxillary dentition was analyzed. Two coordinate systems were established for reference. Global coordinate system: the X-axis represented the coronal direction (positive left, negative right); the Y-axis represented the sagittal direction (positive posterior, negative anterior); and the Z-axis represented the vertical direction (positive superior, negative inferior). Local coordinate system: the origin was located at the centroid of the tooth; the X-axis represented the mesiodistal direction (positive mesial); the Y-axis represented the labiolingual/buccolingual (palatal) direction (positive lingual/palatal); and the Z-axis represented the vertical direction (positive gingival) (Figure 4). The incisal edge center of each incisor, the cusp tip center of each canine, and the occlusal center of the crowns of premolars and molars were selected as measurement points.

**FIGURE 5:**
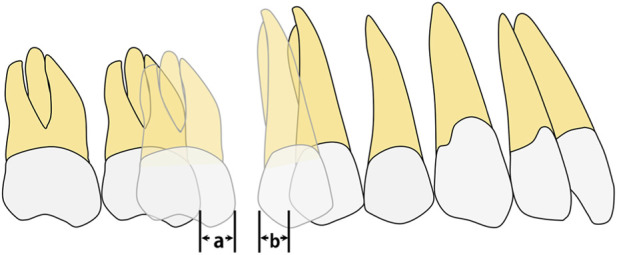
In Modle A, initial closure of the space between the first second molars and the second premolar, including the distal displacement of the second premolar(b), mesial displacement of the first molar(a); U5 ratio, ratio of the second premolar displacement to the initial closure of the interproximal space (a/a+b).

**FIGURE 6:**
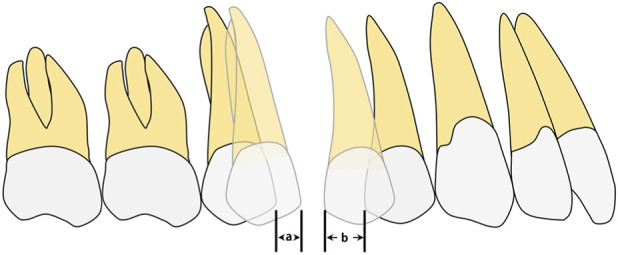
In Modle B, initial closure of the space between the first and second premolars, including the distal displacement of the first premolar(b), mesial displacement of the second premolar(a); U4 ratio, ratio of the first premolar displacement to the initial closure of the interproximal space (a/a+b).

In Models A and B, the following outcomes were measured and analyzed for each condition. (1) Three-dimensional displacement of the maxillary posterior teeth. (2) Three-dimensional displacement of the maxillary anterior teeth. (3) Closure of the space between the first molar and second premolar, including distal displacement of the second premolar, mesial movement of the first molar, total space closure, and the ratio of second premolar distal displacement to total space closure (Figures 5, 6). (4) Analysis of the PDL equivalent stress.

## 3 Results

### 3.1 The second premolar distalization after molar distalization (Model A)

#### 3.1.1 Three-dimensional displacement of the maxillary posterior teeth

**FIGURE 7:**
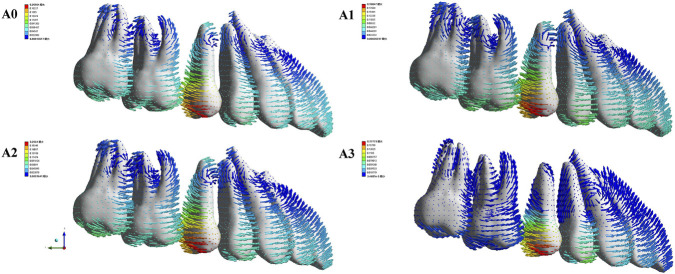
The movement tendency of the maxillary dentition under Modle A during sequential distalization. The coordinate system was based on the entire dentition (global coordinate system). A0, control (conventional aligner design); A1, distal coverage removed at U7; A2, distal coverage removed at U6; and A3, distal coverage removed at both U7 and U6. As shown by the arrows, with the distalization of the maxillary second premolar, the anterior and posterior teeth move in opposite directions due to the exerted reciprocal force. The x-axis represented the coronal plane (+left, −right), the y-axis represented the sagittal plane (+posterior, −anterior), and the z-axis represented the vertical plane (+superior, −inferior).

**FIGURE 8:**
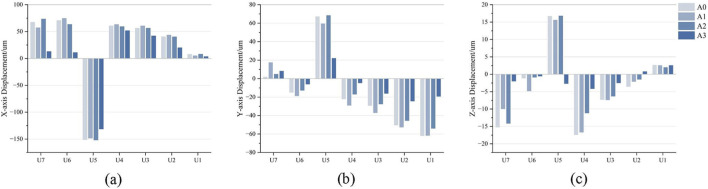
Model A (distalization of the second premolar after molar distalization) displacement trend diagram. **(a)** X-axis: Positive values indicate a mesial inclination tendency, and negative values indicate a distal inclination tendency. **(b)** Y-axis: Positive values indicate a palatal inclination tendency, and negative values indicate a buccal inclination tendency. **(c)** Z-axis: Positive values indicate an intrusion tendency, and negative values indicate an extrusion tendency.

**FIGURE 9:**
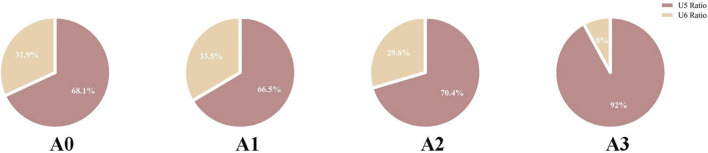
U5 ratio, ratio of the second premolar distal displacement to the initial closure of the interproximal space; U6 ratio, ratio of the first molar mesial displacement to the initial closure of the interproximal space. Compared with A0–A2 (in which the U6 ratio remained ∼30%), A3 markedly reduced the U6 ratio to 8% and increased the U5 ratio to 92%, indicating that the design in A3 substantially decreased the mesial contribution of the already distalized maxillary first molar.

After loading different aligner conditions, all groups showed distal movement of U5, mesial and buccal movement of U4 and U6, and mesial and lingual movement of U7 (Figure 7). In the mesiodistal direction, U5 moved distally in all groups, and the amount of distalization was reduced in A1–A3 compared with the control group. U6 moved mesially in all four conditions, with the greatest mesial movement in A1 and the smallest in A3 (Figure 8). For the space closure between U5 and U6, the contribution of U5 distal displacement to total gap reduction was 68.08%, 66.48%, 70.44%, and 92.01% in A0–A3, respectively, with the highest in A3 (Figure 9). In the buccolingual direction, U7 showed lingual tipping in all groups, whereas U6 showed buccal tipping in all groups. Compared with the control group (A0), the lingual tipping displacement of U7 increased in the distal-end design groups (A1–A3), with the largest increase in A1 and the second largest in A3. This may be because removal of aligner coverage on the distal surface of U7 compromises retention of the second molar. In the vertical direction, U4, U6, and U7 showed extrusion in all conditions, with the smallest extrusion observed in A3 (Figure 8).

#### 3.1.2 Three-dimensional displacement of the maxillary anterior teeth

After loading different aligner conditions, U1-U2 moved mesially and labially in all groups; U3 extruded, U1 intruded, and the maxillary U2 extruded in A0–A2 but intruded in A3 (Figure 7). In mesiodistal direction, A3 showed the smallest mesial movement of the maxillary anterior teeth. In A1, U3 and U2 exhibited the greatest mesial movement. In buccolingual direction, labial tipping displacement of the maxillary anterior teeth decreased in A3. In vertical direction, U1 showed intrusion in all groups, whereas U3 showed extrusion in all groups (Figure 8).

#### 3.1.3 Analysis of periodontal ligament equivalent stress

To further elucidate the biomechanical mechanism of tooth movement, we analyzed the equivalent stress in the periodontal ligament during sequential molar distalization. The maximum PDL equivalent stress consistently occurred in U5. The PDL stress of U6 increased markedly in A1 and was even higher than in A0, whereas it was lowest in A3. These results indicate that during second premolar distalization, removing only the distal-surface coverage at U7 increases the load on U6, which is unfavorable for maintaining the existing space between U5 and U6 (Table 3).

**TABLE 3:** Modle A PDL Von-Mises stress/MPA.

Tooth	A0	A1	A2	A3	B0	B1	B2	B3	B4
U7	0.058	0.062	0.072	0.024	0.041	0.035	0.059	0.049	0.065
U6	0.052	0.054	0.047	0.035	0.043	0.044	0.046	0.048	0.097
U5	0.301	0.288	0.304	0.024	0.064	0.070	0.059	0.059	0.103
U4	0.072	0.080	0.057	0.014	0.266	0.258	0.264	0.263	0.271
U3	0.072	0.087	0.068	0.050	0.071	0.073	0.067	0.067	0.080
U2	0.040	0.049	0.035	0.018	0.060	0.063	0.059	0.057	0.056
U1	0.025	0.025	0.022	0.051	0.032	0.032	0.031	0.031	0.029

### 3.2 The first premolar distalization after distalization of the molars and second premolar (Model B)

#### 3.2.1 Three-dimensional displacement of the maxillary posterior teeth

**FIGURE 10:**
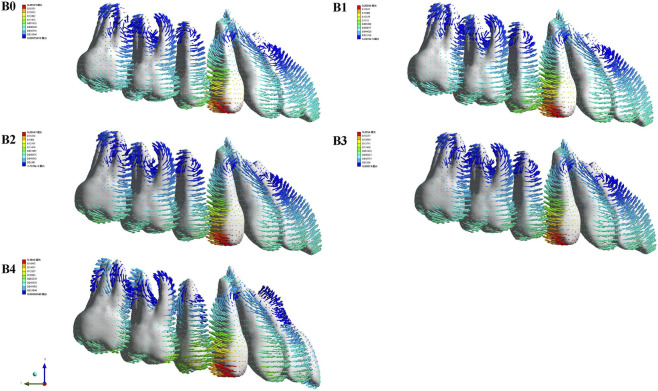
The movement tendency of the maxillary dentition under Modle B during sequential distalization. The coordinate system was based on the entire dentition (global coordinate system). B0, control (conventional aligner design); B1, distal coverage removed at U7; B2, distal coverage removed at U5 and U6; B3, distal coverage removed only at U5; and B4, distal coverage removed at U7, U6, and U5. As shown by the arrows, with the distalization of the maxillary first premolar, the anterior and posterior teeth move in opposite directions due to the exerted reciprocal force. The x-axis represented the coronal plane (+left, −right), the y-axis represented the sagittal plane (+posterior, −anterior), and the z-axis represented the vertical plane (+superior, −inferior).

**FIGURE 11:**
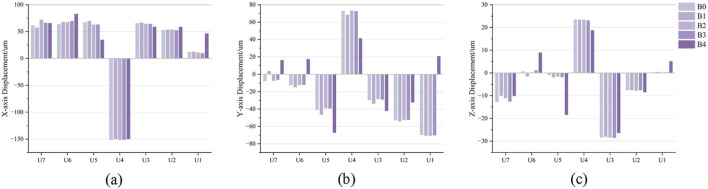
Modle B (In first premolar distalization after distalization of the molars and second premolar) displacement trend diagram. **(a)** X-axis: Positive values indicate a mesial inclination tendency, and negative values indicate a distal inclination tendency. **(b)** Y-axis: Positive values indicate a palatal inclination tendency, and negative values indicate a buccal inclination tendency. **(c)** Z-axis: Positive values indicate an intrusion tendency, and negative values indicate an extrusion tendency.

**FIGURE 12:**
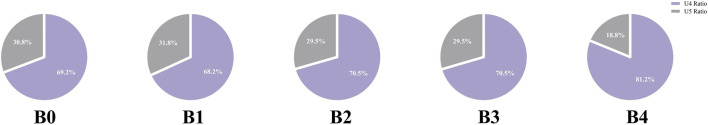
U4 ratio, ratio of the first premolar distal displacement to the initial closure of the interproximal space; U5 ratio, ratio of the second premolar mesial displacement to the initial closure of the interproximal space. Relative to B0–B3 (with a U5 ratio ∼30%), B4 reduced the U5 ratio to 18.8% and increased the U4 ratio to 81.2%, suggesting a marked reduction in the mesial contribution of the already distalized maxillary second premolar.

After loading different aligner conditions, all groups showed distal movement of the U4; U5 moved mesially and buccally in all groups; and U6 and U7 moved mesially, with lingual movement of both molars in B3 (Figure 10). In the mesiodistal direction, U4 moved distally in all groups, and the distalization amount was reduced in B1–B4 compared with the control, with the smallest distalization in B1. U5 moved mesially in all four conditions, with mesial movement ranked as B1 > B0 > B3 > B2 > B4 (Figure 11). Regarding the component ratio of space closure between U4 and U5, the proportion attributable to U4 distal movement was 69.22%, 68.15%, 70.55%, 70.47%, and 81.19% in B0–B4, respectively. The highest contribution of U4 distal movement was observed in B4 (Figure 12). In the buccolingual direction, U6 and U7 showed lingual tipping in B4, and U7 also showed lingual tipping in B1, suggesting that distal non-coverage of the aligner may compromise aligner retention. In the vertical direction, extrusion of U7 ranked from greatest to least as B1 < B4 < B2 < B3 < B0.

#### 3.2.2 Three-dimensional displacement of the maxillary anterior teeth

After loading different aligner conditions, the maxillary anterior teeth moved mesially in all groups, and U3 and U2 moved labially and extruded (Figure 10). In mesiodistal direction, mesial movement of U3 and U2 increased in B1. In buccolingual direction, the increase in labial tipping of U3 was greatest in B4. In vertical direction, U1 showed intrusion in all groups, whereas U2 and U3 showed extrusion in all groups (Figure 11).

#### 3.2.3 Analysis of periodontal ligament equivalent stress

The maximum PDL stress in each group occurred at U4. The largest PDL equivalent stress at U3 was observed in B4, which may be because aligner deformation occurred between U3 and U4 during distalization of U4, resulting in a greater reciprocal force on U3. These formatting styles are meant as a guide, as long as the heading levels are clear, Frontiers style will be applied during typesetting (Table 3).

## 4 Discussion

As full-coverage appliances, clear aligners can control tooth movement more precisely and may provide better vertical control (Caruso et al., 2019). Previous studies have reported that sequential distalization of the maxillary dentition with clear aligners can achieve rates as high as 87%. However, recent studies have shown that, when the entire stage of sequential molar distalization is assessed, the final achievement rate is only 36.48% for the first molar and 41.94% for the second molar (Li et al., 2023).

Ideally, after the second molar has reached its planned distal position, the space between the first molar and the second molar should be occupied by distal movement of the first molar. However, because clear aligners are full-coverage appliances, when the first molar is pushed distally and the space between the second premolar and the first molar increases, the space between the first molar and the second molar inevitably decreases. In this situation, the distal surface of the second molar receives a mesially directed reciprocal force, resulting in mesial movement (relapse). Song et al. using an *in vitro* mechanical experiment, reported that during distalization of the first molar and premolars, the second molar, as the only posterior anchorage unit at the distal end of the arch, bears an equivalent reciprocal force toward mesial movement, thereby creating a risk of mesial relapse (Song et al., 2025). Liu et al. using three-dimensional finite element analysis, found that after the second molar was displaced 2 mm distally to the target position, it showed a tendency for mesial movement under the interaction force generated during first molar distalization, which also supports the presence of relapse (Liu et al., 2023). Clinically, maxillary distalization is commonly combined with miniscrew traction or intermaxillary Class II elastics (Gomez et al., 2015). However, Ren et al. reported that during maxillary molar distalization with clear aligners, even with traction between a buccal miniscrew and the canine, the distalization efficiency of the second molar continued to decrease as subsequent teeth were moved distally. Therefore, skeletal anchorage may help protect anterior anchorage, but it may not completely prevent mesial movement of molars that have already reached the planned positions (Ren et al., 2018). Similarly, relapse may also occur during maxillary premolar distalization, where molars that have already been distalized may move mesially, thereby reducing the overall efficiency of maxillary sequential distalization. If the space gained by sequential distalization is continuously consumed by mesial movement of already distalized teeth, it may substantially compromise anterior alignment or retraction and hinder achievement of the treatment objectives.

Therefore, this study aimed to investigate the premolar distalization stages of sequential distalization by applying different distal-end aligner designs, removing distal-surface coverage of teeth that had already reached the planned positions, to protect posterior anchorage, maintain the space gained during molar distalization, and improve overall sequential distalization efficiency. In this study, three-dimensional finite element models were constructed for the premolar distalization stages with different distal-end aligner designs. The three-dimensional displacement of the maxillary teeth and PDL equivalent stress were measured under different conditions to provide new insights for clinical practice.

### 4.1 In second premolar distalization after molar distalization (Model A)

Based on the component ratio of space reduction between U5 and U6, the contribution of U5 distal movement ranked as A3 > A2 > A0 > A1, with values of 92.01%, 70.44%, 68.08%, and 66.48%, respectively. A3 showed the highest proportion of U5 distal movement, reaching 92.006%. These findings indicate that during maxillary second premolar distalization, removing distal-side aligner coverage on already distalized U6 and U7 reduces the mesially directed reciprocal force on the distalized molars, thereby markedly decreasing their mesial movement and preserving posterior anchorage. It should be noted that A3 showed reduced three-dimensional displacement across the maxillary dentition, possibly due to decreased retention after distal non-coverage leading to aligner dislodgement. Notably, aligner dislodgement or reduced retention are now presented as plausible interpretations based on the simulated force–displacement patterns rather than as directly modeled phenomena. Clinically, this effect may be mitigated by using different attachment combinations on teeth that have reached their planned positions (e.g., adding lingual attachments) or by increasing wear time.

In groups A0–A3, the maxillary anterior teeth showed labial inclination. Clinically, for Angle Class II division 2 patients, this reciprocal effect may be beneficilaIn contrast, for Angle Class II division 1 patients, strategies to protect anterior anchorage are required. Various approaches have been proposed to protect anterior anchorage, such as intermaxillary Class II elastics, miniscrew traction, or preset lingual crown torque of the maxillary anterior teeth.

Regarding arch width, distalization of the maxillary second premolar induced buccal tipping of U6 and U7, thereby increasing posterior arch width. This may be because the dental arch is curved, and tooth movement is not purely sagittal; the corresponding aligner region undergoes deformation in both sagittal and transverse directions. In addition, because posterior contact points are located closer to the buccal side, aligner forces may be transmitted through interproximal contacts. Clinically, negative lingual crown torque may be added to the maxillary molars. During follow-up visits, clinicians should monitor changes in both anterior and posterior occlusal relationships and ensure coordination of maxillary and mandibular arch forms. Vertically, clinicians generally aim to avoid extrusion of the maxillary posterior teeth. This extrusion may cause clockwise rotation of the occlusal plane and an increase in lower facial height. Particularly in high-angle patients, these changes may worsen facial esthetics and may aggravate mandibular retrusion, potentially affecting respiration. In Model A, A3 showed the lowest tendency for molar extrusion. This suggests that distal non-coverage of the aligner may provide better control of vertical anchorage.

### 4.2 In first premolar distalization after distalization of the molars and second premolar (Model B)

B4 showed the smallest mesial movement of U5, and the highest contribution of U4 distal movement to space reduction between U4 and U5 (81.19%). These findings indicate that during maxillary first premolar distalization, distal non-coverage of the aligner can also protect posterior anchorage and reduce mesial relapse of already distalized teeth. Compared with Model A, the differences in total maxillary displacement and individual tooth displacement among conditions were less pronounced in Model B. Possibly because during first premolar distalization, U5, U6, and U7 had already been distalized and acted as a more stable anchorage unit against reciprocal forces.

It should be noted that reduced mesial relapse of already-positioned teeth was observed in both A3 and B4, which may reflect the combined influence of two mechanisms. First, a true stability gain may occur: removing distal aligner coverage can decrease mesially directed forces acting on already-positioned teeth, thereby improving the stability of distalization and reducing relapse. Second, an apparent reduction in relapse may arise from efficiency loss: compromising the structural integrity of the aligner can weaken retention and reduce overall movement efficiency across the dentition, thereby creating the illusion of less relapse in already-positioned teeth. Consistently, analysis of the space-closure ratio indicates that the A3 and B4 designs substantially reduce the proportion of space closure attributable to mesial movement of the already-positioned teeth. This finding suggests that teeth that have already reached the intended distal position can be maintained more effectively in subsequent stages, without compromising the expression of other programmed movements. Clinically, fewer “distalize–rebound–re-distalize” cycles translate into more controllable and durable outcomes. Reduced relapse without an obvious efficiency penalty is therefore more consistent with a genuine stability benefit. Future studies could combine the distal non-coverage with attachments of different morphologies, locations, and numbers to identify an integrated strategy that preserves retention while minimizing relapse.

Notably, A1 and B1 were designed with distal non-coverage only at U7. This design was based on the assumption that U7 has the largest aligner coverage area and thus might exert the greatest influence on posterior anchorage. However, our results showed that A1 had the lowest contribution of U5 distal displacement, even lower than the control. A similar trend was observed in B1. Therefore, during premolar distalization, removing only the distal coverage of U7 may shift anchorage demand from the second molar to the anterior teeth and adjacent posterior teeth, increasing mesial movement of the mesial teeth, consuming the space gained in earlier stages, and ultimately compromising maxillary anterior alignment or retraction.

In Model B, maxillary molars showed buccal tipping in B0, B1, B2, and B3, whereas palatal tipping was observed in B4. Meanwhile, B4 showed the greatest buccal tipping tendency of U5. These findings suggest that when distal non-coverage is applied to U7, U6 and U5, the marked reduction in aligner coverage area may compromise precise control of the posterior teeth. Future studies may need to combine distal non-coverage with different posterior attachment positions or shapes to maintain posterior aligner retention while reducing mesial relapse of already distalized teeth. Vertically, B4 showed the smallest tendency for extrusion of U7. This suggests that a distal non-coverage design may provide better vertical anchorage control. Meanwhile, B4 showed lingual tipping of U1 accompanied by extrusion. This may be because, after applying distal non-coverage at U7, U6 and U5, deformation of the aligner in the anterior segment generated lingually and occlusally directed forces on the central incisor. Periodontal stress distribution is positively correlated with root resorption and may indicate the risk of root resorption (Han et al., 2005). B4 showed increased canine PDL equivalent stress, and U3 tended to tip buccally. This suggests that canine torque should be closely monitored clinically to avoid bony dehiscence or fenestration.

Because clear aligners are made of thermoplastic resin, their material properties may change intraorally due to temperature, humidity, stress relaxation, and saliva (Dalaie et al., 2021). CA materials in this study were consistently identical to their initial condition, and factors such as stress relaxation and material aging caused by saliva and occlusal forces were not considered. Therefore, the model cannot capture the long-term displacement trends (Gao et al., 2024; Zhang et al., 2024; Elshazly et al., 2023). In addition, three-dimensional finite element analysis reflects only the initial tooth displacement under load and does not account for factors such as alveolar bone remodeling, periodontal tissue remodeling, occlusal forces, and patient compliance, and therefore cannot fully replicate the orthodontic process (Tanne et al., 1987). The finite element model in this study was based on CBCT data from a single patient, which limits the generalizability of the simulation findings to a larger population. This study provides a descriptive biomechanical comparison. Future work should incorporate experimental validation and patient-specific modeling to better establish clinical relevance, with statistical analyses performed where appropriate. In the present simulations, we evaluated the effects of clear aligners alone on the maxillary dentition, without commonly used adjuncts such as intermaxillary elastics or miniscrew-assisted traction. Therefore, future studies should examine these adjunctive traction strategies—alone and in combination—to develop a standardized protocol that can reliably support maxillary sequential distalization. Finally, larger clinical studies and well-documented case series are needed to strengthen the evidence for the distal-end aligner designs evaluated here and to confirm the robustness and reproducibility of our findings.

## 5 Conclusion

Maxillary sequential distalization can be divided into three stages: molar distalization, premolar distalization, and a stage in which the gained space is used for alignment and anterior retraction. This study used three-dimensional finite element analysis to evaluate the influence of different distal-end aligner designs on the success of sequential distalization. The findings were as follows.

1. During second premolar distalization after molars had reached their planned positions, removing distal coverage at U7 and U6 effectively reduced mesial relapse of the distalized molars, thereby preserving posterior anchorage and maintaining the space gained by sequential distalization.
2. During first premolar distalization after the second premolar and molars had reached their planned positions, removing distal coverage at U7, U6and U5 reduced mesial relapse of the two molars and the second premolar
3. After removing distal-end aligner coverage, protection of anterior anchorage should be considered in clinical practice.

## Data Availability

The original contributions presented in the study are included in the article/supplementary material, further inquiries can be directed to the corresponding author.
